# Updated efficacy and toxicity of treatment with the anti-CTLA-4 antibody ipilimumab in metastatic melanoma patients previously treated with anti-PD-1 therapy

**DOI:** 10.1186/2051-1426-3-S2-P126

**Published:** 2015-11-04

**Authors:** Prashanth Prithviraj, Grant McArthur, Victoria Atkinson, Phillip Parente, Miles Andrews, Sagun Parakh, Michael Millward, Jonathan Cebon, Oliver Klein

**Affiliations:** 1 Medical Oncology, Austin Health, Heidelberg, Australia; 2 Peter MacCallum Cancer Centre, East Melbourne, Australia; 3 Princess Alexandra Hospital, Woolloongabba, Australia; 4 Eastern Health Clinical School, Box Hill Hospital, Box Hill, Australia; 5 Sir Charles Gairdner Hospital, Nedlands, Australia; 6 Olivia Newton-John Cancer Research Institute, Heidelberg, Australia

## Background

Immunotherapy with anti-CTLA-4 and anti-PD-1 antibodies has demonstrated overall survival benefits in patients (pts) with metastatic melanoma (MM) compared to previous standard therapy. Two randomised clinical trials indicate that combined anti-CTLA-4 and anti-PD-1 antibody therapy increases the response rate compared to single agent treatment, but is associated with increased toxicity [1,2]. Both efficacy and toxicity of anti-PD-1 therapy appear independent of prior treatment with the anti-CTLA-4 antibody ipilimumab. To date, only limited evidence exists regarding the efficacy and toxicity of Ipilimumab in pts that have progressed on treatment with an anti-PD-1 agent.

## Methods

We retrospectively identified pts with MM who received anti-PD-1 therapy (Nivolumab/Pembrolizumab) and were subsequently treated with ipilimumab. Ipilimumab was administered at a dose of 3mg/kg every three weeks for (up to) four doses and response assessed by CT scan 4-6 weeks after the last dose. Efficacy and toxicity outcomes were determined from clinical records.

## Results

The median age was 53 years with all pts having stage IVc disease and 4 pts (33%) with an elevated LDH at commencement of Ipilimumab dosing. The median time between the last dose of anti-PD-1 therapy and the commencement of Ipilimumab was 8 months (range 2-14 months). After a median follow-up of over 6 months, 1 patient (8%) achieved a partial remission as their best response to anti-PD-1 therapy with an additional 6 (50%) having stable disease. Five patients (42%) received all four doses of Ipilimumab. Two patients (17%) achieved an objective response to ipilimumab with another having prolonged stable disease. Four patients experienced grade 3/4 immune-related adverse events (irAE) including colitis (n=3) and pneumonitis (n=1).

## Conclusions

Ipilimumab therapy can induce responses in patients who have failed treatment with an anti-PD-1 antibody. The response rate and clinical benefit rate appears similar compared to outcomes in pts who have not received prior anti-PD-1 antibody therapy. Although cases of severe and/or unusual irAEs such as pneumonitis have been observed, an analysis of a larger patient cohort will be required to test the significance of these observations.
